# Alpha-amylase as the culprit in an occupational mealworm allergy case

**DOI:** 10.3389/falgy.2022.992195

**Published:** 2022-08-30

**Authors:** Eva Ganseman, Toon Ieven, Glynis Frans, Lieve Coorevits, Noëmie Pörtner, Erik Martens, Dominique MA Bullens, Rik Schrijvers, Christine Breynaert, Paul Proost

**Affiliations:** ^1^ Laboratory of Molecular Immunology, Rega Institute, Department of Microbiology, Immunology and Transplantation, KU Leuven, Leuven, Belgium; ^2^ Allergy and Clinical Immunology Research Group, Department of Microbiology, Immunology and Transplantation, KU Leuven, Leuven, Belgium; ^3^ Department of General Internal Medicine, Allergy and Clinical Immunology, University Hospitals Leuven, Leuven, Belgium; ^4^ Laboratory Medicine, University Hospitals Leuven, Leuven, Belgium; ^5^ Laboratory of Immunobiology, Rega Institute, Department of Microbiology, Immunology and Transplantation, KU Leuven, Leuven, Belgium; ^6^ Department of Pediatrics, University Hospitals Leuven, Leuven, Belgium

**Keywords:** mealworm, occupational asthma, alpha-amylase, edible insect, *tenebrio molitor*

## Abstract

**Background:**

Occupational allergy has been described in employees working in contact with mealworms in pet stores, live fish bait or infested stored grains and recently, in mealworm farming for animal feed and human consumption. Mealworm allergens linked to occupational allergy are troponin C, cockroach-like allergen, tropomyosin, arginine kinase, early-staged encapsulation inducing- and larval cuticle proteins.

**Objective:**

We report a case of occupational mealworm allergy and studied the culprit component.

**Methods:**

Diagnosis was done by skin prick, specific IgE, basophil activation and lung function testing. Allergen purification was performed by anion-exchange chromatography and immunoblotting with patient IgE. Allergens were identified by in-gel trypsin digest and tandem mass spectrometry. Allergenicity and specificity further confirmed by IgE inhibition and passive basophil activation experiments.

**Results:**

We describe a new case of occupational mealworm allergy in a laboratory worker, with sensitization to different developmental stages and derivates of the mealworm. In basophil activation tests, the majority of patient's basophils (69%–91%) degranulated upon stimulation with the lowest concentration of mealworm extracts (0.16 µg/ml). Despite strong sensitization to mites, the patient did not show cross-reactivity to other insects. We were able to identify alpha-amylase as the main allergen and through inhibition experiments, we demonstrated that low amounts (0.1 µg/ml) of this allergen could strongly inhibit mealworm specific IgE by 79.1%. Moreover, passive BAT experiments demonstrated the IgE-alpha-amylase interaction to be functional, inducing up to 25.5% degranulation in healthy donor basophils.

**Conclusion:**

Alpha-amylase can be identified as the responsible allergen in this specific case of occupational mealworm allergy.

## Introduction

The yellow mealworm or *Tenebrio molitor* is part of the *Tenebrionidae* or black beetle family. Mealworm farming has been of interest on a rather small scale in the context of fish bait and pet feed (1). However, due to their limited need for water, feed and land area combined with a beneficial protein content, the focus is shifting towards human consumption as well (2). Recently, the European Union (EU) approved the use of mealworms for human consumption (3). Despite EU approval, mealworm-containing food should be labeled with a warning for patients with crustacean and house dust mite allergy, as cross-reactivity to mealworm and other insects can be clinically relevant (4, 5). Besides concerns related to allergic reactions upon consumption of mealworm-containing food, it has been shown that close occupational contact with mealworm may be associated with allergy. This has been reported in fishers (6–8), pet store employees (9) or employees exposed to flour (10, 11) where mealworms can be a pest. More recently, two cases of occupational allergy in employees involved in the production of insect flour for human consumption, were reported (12). Primary mealworm allergy can be caused by pan-allergens, common to different insect species, such as arginine kinase, tropomyosin, troponin C, myosin light and heavy chain or by novel mealworm-specific allergens such as larval cuticle protein A1A, A2B and A3A (13, 14). Here, we describe a new case of occupational mealworm allergy in a laboratory worker involved in the optimization of mealworm farming. Considering mealworm farming is an emerging industry and commercial (component-resolved) diagnostics are lacking, apart from a mealworm specific IgE test, we decided to investigate this case thoroughly and we identified a novel underlying culprit allergen.

## Methods

### Extract preparation

All insects were collected fresh and immediately frozen at −20°C. For extract preparation, insects were blended and subsequently dissolved in phosphate buffered saline (PBS, Lonza, Basel, Switzerland) at 15%–20% (w/v) depending on the structure of the material. Extracts were shaken for 1–2 h at room temperature and centrifuged at 700 g for 10 min at 4°C. After centrifugation, the supernatant was filtered through a 0.22 µM filter (VWR, Haasrode, Belgium) and frozen at −20°C until further use. House dust mite extract was purchased from Greer laboratories (Lenoir, USA), dissolved in PBS, filtered through a 0.22 µM filter and stored at −20°C. Protein quantification was achieved by a bicinchoninic acid assay (BCA) using the Pierce™ BCA Protein Assay Kit (Thermofisher, Waltham, USA) according to the instructions of the manufacturer.

### Specific IgE and inhibition experiments

Specific IgE tests were performed *via* an ImmunoCAP fluorescence enzyme immunoassay on a Phadia 1,000 analyzer (Thermofisher). The following specific IgE tests were performed: mealworm (o211), birch pollen (t3), Bet v 1 (t215), Timothy grass (g6), Der *p* 1 (d202), Der *p* 2 (d203), Der *p* 10 (d205), Der *p* 23 (d209), *Dermatophagoides farinae* (d2), *Euroglyphus maynei* (d74), *Acarus siro* (d70), *Tyrophagus putrescentiae* (d72), cockroach (i6) and shrimp (f24). In inhibition experiments, the stimulus was added to serum 1:4, followed by overnight incubation at 4°C. As a baseline value, a condition where we added buffer (PBS or purification solvents), was included. This baseline level of specific IgE was used to calculate a percentage of inhibition.

### Basophil activation test (BAT) and passive BAT

Whole blood samples were drawn using lithium/heparin tubes (BD, New Jersey, USA) and experiments were started within 1 h. Each stimulus was diluted in basophil stimulation buffer containing 20 mM HEPES, 133 mM NaCl, 5 mM KCl, 7.5 mM CaCl_2_, 3.5 mM MgCl_2_, 1 mg/ml human serum albumin (HSA), 0.5 mM glucose and 60 ng/ml interleukin (IL)-3 (PeproTech, Cranbury, USA) at pH 7.4. Stimulation of basophils was induced by adding 30 µl of stimulus in 150 µl whole blood followed by incubation for 25 min at 37°C. As IgE-mediated control, we used polyclonal goat anti-human IgE antibodies (aIgE, 50 ng/ml, Sigma-Aldrich, Missouri, USA) and N-formyl-methionyl-leucyl-phenylalanine (fMLF, 40 nM, Sigma-Aldrich) as IgE- and non-IgE-mediated positive controls, respectively. Basophil stimulation buffer was used as a negative control. The reaction was stopped at 4°C for 5 min followed by incubation with fluorochrome-linked antibodies for 25 min at 4°C including anti-CD123-PE (111 ng/ml), anti-HLA-DR-AlexaFluor 647 (111 ng/ml) and anti-CD63-FITC (444 ng/ml) (Biolegend, San Diego, USA). Subsequently, red blood cells were lysed by adding 2 ml of lysis buffer (BD) for 10 min at room temperature in the dark. After washing with 2 ml of PBS, cells were fixed in 1% paraformaldehyde. Basophils were analyzed on an LSRFortessa flow cytometer equipped with FACSDiva software (BD). Basophils were identified as CD123^+^ and HLA-DR^−^ cells and degranulation was defined by detection of CD63 expression on basophils.

For passive BAT, peripheral blood mononuclear cells (PBMCs, 5.10^6^ cells) of a healthy nonallergic donor were isolated through density gradient centrifugation over a ficoll gradient (LymphoPrep, Stem Cell Technologies, Vancouver, Canada). PBMCs were then stripped of surface-bound IgE by adding acidic stripping buffer containing 0.9% NaCl, 50 mM KCl and 13.4 mM lactic acid. After incubation on ice for 5 min, neutralization buffer was added, containing 0.5% HSA, 12 mM Tris-HCl (pH 8) in Roswell Park Memorial Institute (RPMI) 1,640 Medium (Thermofisher). Immediately after neutralization, cells were centrifuged at 1,200 g for 5 min at 4°C and washed in 2 ml PBS. Cells were then incubated for 1 h at 37°C with serum of the patient, diluted 1:1 in either PBS or an inhibitory stimulus, pre-incubated overnight at 4°C. After incubation of cells with patient serum, approximately 2.5 × 10^5^ to 5.10^5^ sensitized donor cells were stimulated and stained per condition, identical to the standard basophil activation test but without the red blood cell lysing step.

### Immunoblotting and silver stain

Extracts or purified allergens were diluted in sample buffer containing 0.125 mM Tris (Sigma-Aldrich), 4% sodium dodecyl sulfate (SDS, Roth, Karlsruhe, Germany), 20% glycerol (Acros, Geel, Belgium) and 10% 2-mercaptoethanol (Bio-Rad, Hercules, USA). Diluted samples were loaded on 10% Tris-glycine gels (Novex, Thermofisher) and ran in running buffer containing 192 mM glycine (VWR chemicals), 25 mM Tris and 0.1% SDS at pH 7.4. Proteins were transferred to polyvinylidene fluoride (PVDF) membranes using transfer buffer (Bio-Rad) and membranes were blocked for 1 h in 5% (w/v) bovine serum albumin (BSA, Sigma-Aldrich) in wash buffer containing 20 mM Tris, 150 mM NaCl, 0.1% (w/v) Tween 20 (Merck, Kenilworth, Germany) at room temperature. After three wash steps of 5 min at room temperature, membranes were incubated overnight at 4°C in patient serum diluted 1:10 in blocking buffer. After three wash steps, membranes were incubated with mouse anti-human IgE antibody (1:1,000, GeneTex, Irvina, USA). Excess antibody was washed away (5 times) and membranes were finally incubated for 1 h at room temperature with goat anti-mouse IgE antibody coupled to horseradish peroxide (1:10^4^, Dako, Jena, Germany). After washing, SuperSignal™ West Femto substrate was used to develop western blots (Thermofisher). For silver stains, the same SDS-page protocol was followed. Silver staining was performed using SilverQuest™ Silver Staining Kit (Thermofisher) according to the instructions of the manufacturer.

### Purification and identification of allergens

Purification of potential allergens from dried mealworm extract, was performed by anion exchange chromatography. Separation was done using a MonoQ column (Cytiva, Marlborough, USA) and an AKTA purifier (GE Healthcare, Chicago, USA). The loading buffer consisted of 20 mM Tris at pH 8 and elution buffer consisted of 20 mM Tris and 1M NaCl at pH 8. Elution fractions were screened by SDS-page (10% Tris glycine gels, Thermofisher) and stained with silver (Invitrogen, Waltham, USA) or Coomassie blue (InstantBlue™, Abcam, Cambridge, UK). Potential candidates were further tested in immunoblot experiments using patient samples, as described before. Allergens were finally identified by in-gel trypsin digestion and nano scale liquid chromatography followed by tandem mass spectrometry by AlphaLyse (Odense, Denmark).

### Ethical approval

Collection of blood samples and clinical data was performed in accordance with a prospective study protocol approved by the Ethics Committee Research UZ/KU Leuven (study no° S60734 and S65293). The patient and healthy controls provided written informed consent prior to participation.

## Results

### Case report of a 34-year-old female laboratory worker suffering from occupational asthma

A 34-year-old female laboratory worker presented to the allergy department of the University Hospitals Leuven due to gradually worsening asthmatic symptoms, presumably upon occupational contact with two insect species: *Tenebrio molitor* (yellow mealworm) and *Hermetia illucens* (black soldier fly). Initial symptoms included ocular pruritus and rhinorrhea upon close contact with insects, but developed into coughing and wheezing later on. Symptoms improved during weekends or holidays, suggesting an occupational factor. Moreover, the patient attempted consumption of mealworm once and experienced immediate tingling of the mouth, without swelling of lips or tongue.

**Figure 1:**
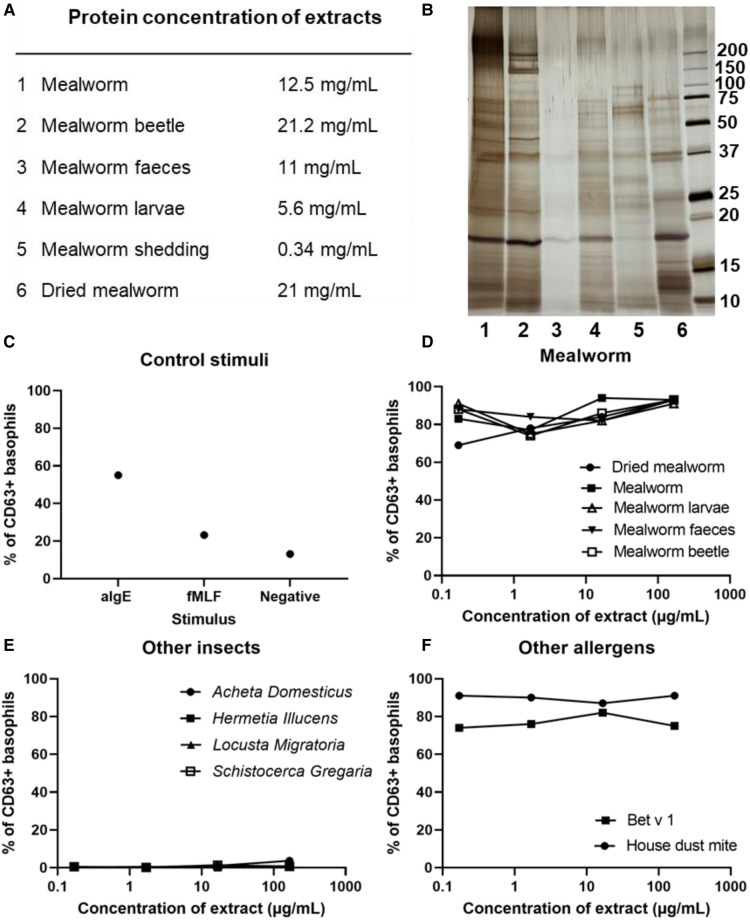
Confirmation of mealworm allergy by Basophil activation tests. (**A**) Protein concentrations of in-house generated mealworm extracts, measured by BCA. (**B**) SDS-page/silver stain of mealworm extracts, 5 µg of each extract loaded. x-axis in panels (**D**–**F**): Log-transformed protein concentration range of different extracts used, Y-axis in panels **C**–**F**: % of CD63+ basophils i.e. % degranulated basophils. (**C**) Positive controls: aIgE: anti-IgE antibody, fMLF: N-formyl-methionine-leucyl-phenylalanine; Negative control: basophil stimulation buffer. (**D**) Different extracts derived from mealworm, (**E**) insect extracts, and (**F**) Bet v 1 and house dust mite (Greer).

No commercial extracts were available for allergy diagnosis, leading to in-house generation of water soluble protein extracts of mealworm (Figures 1A,B) and other insects. To assess the protein content of each extract, an SDS-page was ran and silver-stained (Figure 1B), showing that the protein quantification of mealworm faeces was strongly overestimated. All other extracts show a wide variety of protein bands, with a major protein of approximately 17 kDa present in all extracts. The in-house generated extracts were used in skin prick tests (SPT, Supplementary Table) and tests were positive for all different developmental stages of the mealworm (larvae, worm and beetle) and derivatives (shedded skin and faeces). Likewise, a SPT for freeze-dried mealworm, a variety intended for human consumption, was positive. SPT for black soldier fly were all negative, including different developmental stages (worm and fly) and derivatives (shedded skin and faeces). SPT with other insects (*Schistocerca gregaria*, *Locusta migratoria*, *Blaptica dubia* and *Acheta domesticus*) were all negative. SPT were positive for multiple aero-allergens including grass, rye, alder and birch pollen and both European and American house dust mite. Specific IgE (sIgE, Supplementary Table) confirmed sensitization to mealworm, various pollen (birch and timothy grass) and mites (*Dermatophagoides farinae*, *Euroglyphus maynei*, *Acarus siro* and *Tyrophagus putrescentiae*). Component-resolved diagnostics demonstrated presence of specific IgE towards Der p 1, Der p 2, Der p 23 but not to the potentially cross-reactive allergen Der *p* 10 (tropomyosin). The house dust mite sensitization was already present two years before the onset of insect-related symptoms, but house dust mite-related symptoms were controlled by preventive dust measures. There were no signs of seasonal rhinoconjunctivitis, despite pollen sensitization.

Baseline spirometry was normal, but reversible airway obstruction was observed, as the forced expiratory volume in 1 s (FEV1) improved significantly by 17.4% after administration of salbutamol. The fraction of exhaled nitric oxide was elevated to an intermediate level of 57.2 parts per billion. The lung function test results were considered compatible with asthma.

Subsequent basophil activation tests (BAT), demonstrated reactivity to all derivatives of the mealworm in comparable extract concentrations (0.167 µg/ml to 167 µg/ml, Figures 1C,D). Shedded skin of the mealworm was not tested in BAT due to the low protein content of the extract (0.34 mg/ml, Figure 1A). A mealworm BAT was performed in 9 healthy controls (Supplementary Figure S1), showing some degree of non-specific degranulation to high doses of mealworm extract in two controls (HC1 and HC3). Therefore, the two highest concentrations (16.7–167 µg/ml) fail to indicate specific reactions, whereas the two lower concentrations (0.167–1.67 µg/ml) confirm mealworm allergy. BAT for other insects (*Schistocerca gregaria*, *Locusta migratoria*, *Blaptica dubia* and *Acheta domesticus*) were negative for our patient (Figure 1E), corresponding to the SPT. Bet v 1 and house dust mite also elicited basophil degranulation, mirroring SPT and sIgE results (Figure 1F).

### Alpha-amylase as molecular cause of mealworm allergy

**Figure 2:**
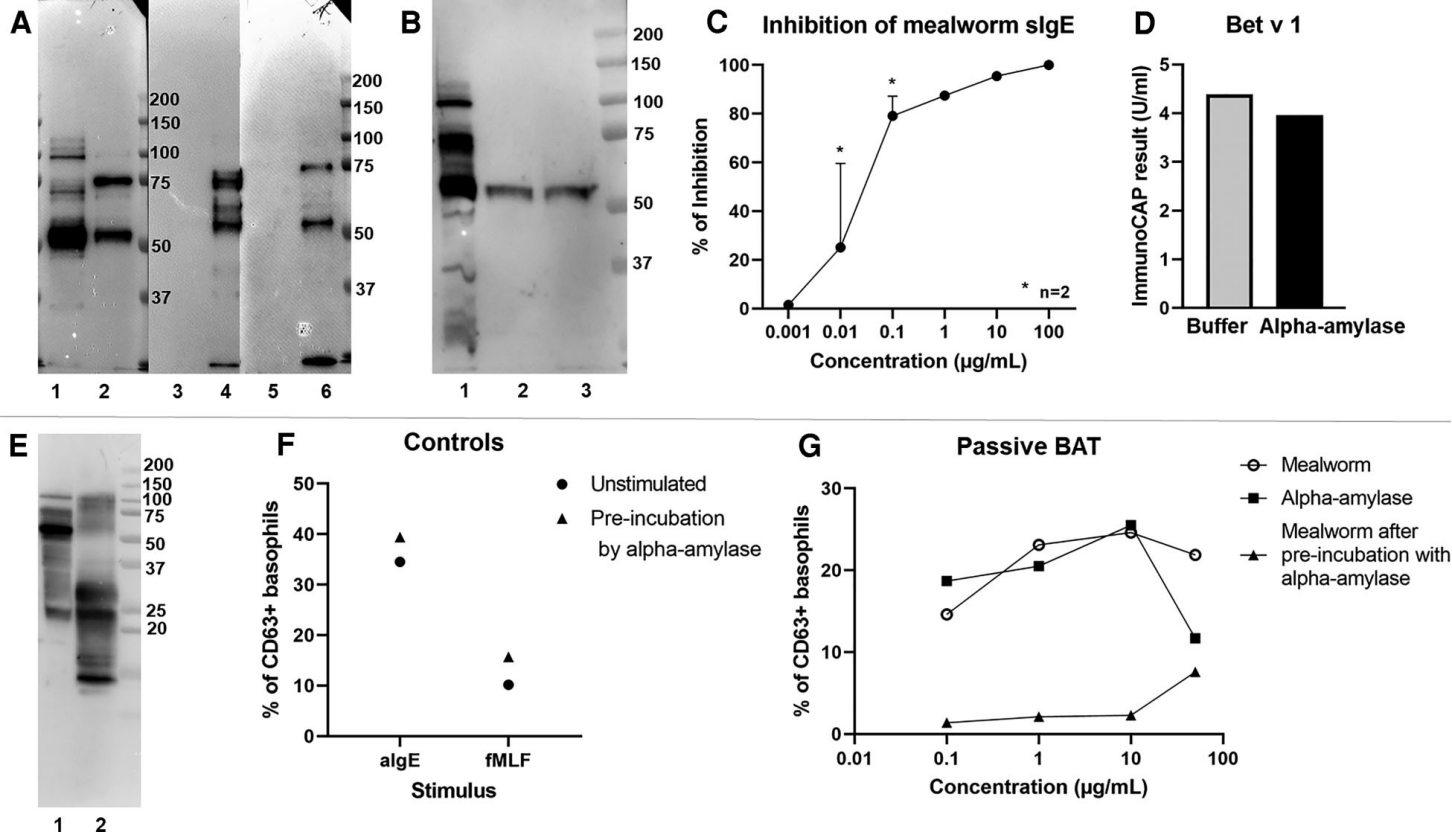
Experimental evidence demonstrating alpha-amylase to be the culprit allergen. (**A**) Immunoblot with serum of the patient and detection for IgE binding. Blotted proteins, 5 µg loaded of each: 1. mealworm; 2. beetle; 3. faeces; 4. larvae; 5. shedded skin; 6. dried mealworm (**B**) Immunoblot with serum of the patient and detection of IgE. Blotted proteins, 5 µg loaded of each: 1. mealworm; 2. pooled ion-exchange fractions E2-E3; 3. pooled ion-exchange fractions E4-E5. (**C**) Measurement of mealworm specific IgE in an ImmunoCAP with pre-incubation of patient serum with alpha-amylase. Y-axis: % of inhibition, x-axis: concentration of alpha-amylase used for pre-incubation; *[for 0.01 and 0.1 *µ*g/ml results from 2 separate experiments are shown as mean + SEM]*. (**D**) Inhibition of Bet v 1 reactivity as unrelated sIgE by pre-incubation of patient serum with alpha-amylase. (**E**) Immunoblot with serum of the patient and detection of IgE. Blotted proteins, 5 µg loaded of each: 1. mealworm; 2. house dust mite (Greer). (**F**) Passive BAT results with control stimuli in baseline sensitization conditions (circles) or after pre-incubation of serum with alpha-amylase 10 µg/ml (triangles), aIgE: anti-IgE antibody, fMLF: N-formyl-methionine-leucyl-phenylalanine, % CD63 + basophils normalized to the negative control (basophil stimulation buffer). (**G**) Passive BAT results after stimulation with mealworm extract (open circles), alpha-amylase (squares) or mealworm extract with pre-incubation of patient serum with alpha-amylase 10 µg/mL (triangles). Y-axis: basophil degranulation represented as % of CD63 + basophils normalized to the negative control (basophil stimulation buffer), x-axis: concentration of stimulus.

Through immunoblotting, we attempted to pinpoint the causative allergen responsible for this occupational allergy. The patient showed the strongest IgE binding to a 55 kDa protein in in-house generated mealworm, beetle, larvae and dried mealworm extracts (Figure 2A). Anion exchange chromatography was performed to separate potential allergens in different fractions, which were subsequently tested in immunoblots using the patient's serum and a secondary anti-IgE reporter antibody. Four fractions were shown to contain the 55 kDa allergen (Figure 2B). In-gel trypsin digest and tandem mass spectrometry identified this protein as alpha-amylase (Supplementary Figure S2). In subsequent inhibition experiments, we pre-incubated the patient's serum with the purified mealworm alpha-amylase (1 ng/ml to 100 µg/ml, Figure 2C), leading to a potent inhibition of sIgE reactivity to mealworm from 0.1 µg/ml of alpha-amylase onward. To make sure that this inhibition was not based on non-specific enzymatic activity, we repeated this experiment with a specific IgE test unrelated to mealworm allergy, i.e. Bet v 1. No significant inhibition of IgE reactivity to Bet v 1 was observed, indicating that the inhibition by alpha-amylase was mealworm-specific (Figure 2D). Moreover, immunoblotting with house dust mite extract (Figure 2E) did not reveal significant binding of patient IgE to full length house dust mite alpha-amylase (57 kDa, P49274, AMY_DERPT) which shares 49% sequence homology with mealworm alpha-amylase (P56634, AMY_TENMO). This suggests the house dust mite and mealworm sensitization in this patient could be based on co-sensitization rather than alpha-amylase cross-reactivity, although we cannot exclude the house dust mite alpha-amylase to be degraded and thus not present at 57 kDa in our immunoblot experiments.

Additionally, we performed passive BATs in which basophils of a healthy non-allergic donor were stripped of their IgE and loaded with the patient's IgE (Figures 2F,G). Up to 25.5% of the passively sensitized donor basophils degranulated upon exposure to 10 µg/ml of both mealworm extract and alpha-amylase, demonstrating the ability of the patient's alpha-amylase-specific IgE to induce effective basophil degranulation. The same experimental set-up was performed in an inhibitory context, where patient serum was pre-incubated overnight with alpha-amylase prior to sensitization of donor basophils. In this experiment, pre-incubation of serum with 10 µg/ml of alpha-amylase did not affect basophil responses to positive control stimuli (Figure 2F) but completely inhibited degranulation after stimulation with up to 10 µg/ml of mealworm extract (Figure 2G).

## Discussion

We described a novel case of mealworm allergy in a laboratory employee, working in a facility involved in the optimization of mealworm farming, presenting with rhino-conjunctivitis and asthma upon mealworm exposure. The patient had detectable mealworm specific IgE in serum and positive SPT to mealworm, beetle, larvae, faeces, dried mealworm and the shedded skin of the mealworm. BAT experiments confirmed the broad sensitization to all developmental stages and derivatives of the insect. Moreover, despite presence of strong sensitization to mites, the patient did not demonstrate any IgE towards tropomyosin, often suggested as a cross-reactive allergen between insects and mites. The role of pan-allergens as culprits for the observed mealworm allergy was further excluded through SPT and BAT experiments which confirmed the absence of cross-sensitization to other insects.

Through immunoblotting, ion-exchange chromatography and in-gel trypsin digest followed by MS/MS analyses, we identified alpha-amylase as the main allergen in this patient. Alpha-amylase is a digestive enzyme of the mealworm and is known to be an allergen in different insect species including mites [Der p 4 and Der f 4, (15, 16)] and cockroaches [Bla g 11 and Per a 11, (17, 18)]. Previously, Verhoeckx et al. (19) predicted mealworm alpha-amylase to be allergenic by homology research and following this, Broeckhoven et al. (20) showed that IgE from house dust mite allergic patients can cross-react with alpha-amylase in immunoblots. Nonetheless, we did not observe binding of our patient's IgE towards full length house dust mite alpha-amylase in immunoblotting. These finding suggest a co-sensitization to mealworm and house dust mite, rather than a cross-sensitization.

Further supporting the allergenicity of mealworm alpha-amylase, we showed that our patient's IgE, when loaded on healthy control basophils, induced degranulation when cross-linked by alpha-amylase. Moreover, alpha-amylase strongly inhibited mealworm specific IgE and reactivity towards mealworm in passive BAT. In summary, our results demonstrate for the first time that alpha-amylase is a potential culprit allergen in occupational mealworm allergy.

## Data Availability

The original contributions presented in the study are included in the article/Supplementary Materials, further inquiries can be directed to the corresponding author/s.
