# Supplementary material for: Alpha-amylase as the culprit in an occupational mealworm allergy case
Source: Front Allergy. 2022 Aug 30;3:992195. doi: 10.3389/falgy.2022.992195 (PMC9468247; doi:10.3389/falgy.2022.992195)
Supplement: Supplementary file 1 [file Data_Sheet_1_v1.pdf]

Supplementary Table 1. Overview of clinical data of the patient.

Skin prick test results		Specific IgE test results (kU/L)	
Saline	-	Mealworm	4.43
Histamine	+	*Dermatophagoides farinae*	8.70
Mealworm	++	Der p 1	1.32
Mealworm - beetle	++	Der p 2	3.13
Mealworm - Shedded skin	++	Der p 10	<0.1
Mealworm – Faeces	++	Der p 23	2.63
Mealworm - Larvae	+	*Euroglyphus maynei*	1.25
Dried mealworm	+	*Acarus siro*	0.52
Black soldier fly	-	*Tyrophagus putrescentiae*	0.34
Black soldier fly – worm	-	German cockroach	<0.1
Black soldier fly – Shedded skin	-	Shrimp	<0.1
Black soldier fly - Faeces	-	Birch pollen	4.17
Blaptica dubia	-	Bet v 1	5.12
Acheta domesticus	-	Timothy grass	5.73
Locusta migratoria	-		
Schistocerca gregaria	-		
Grass pollen	++		
Rye pollen	++		
Alder tree pollen	+		
Birch pollen	+		
Dermatophagoides pteronyssinus	+		
Dermatophagoides farinae	+		
Shrimp	-		
