# Supplementary figures and images for: Alpha-amylase as the culprit in an occupational mealworm allergy case
Source: Front Allergy. 2022 Aug 30;3:992195. doi: 10.3389/falgy.2022.992195 (PMC9468247; doi:10.3389/falgy.2022.992195)

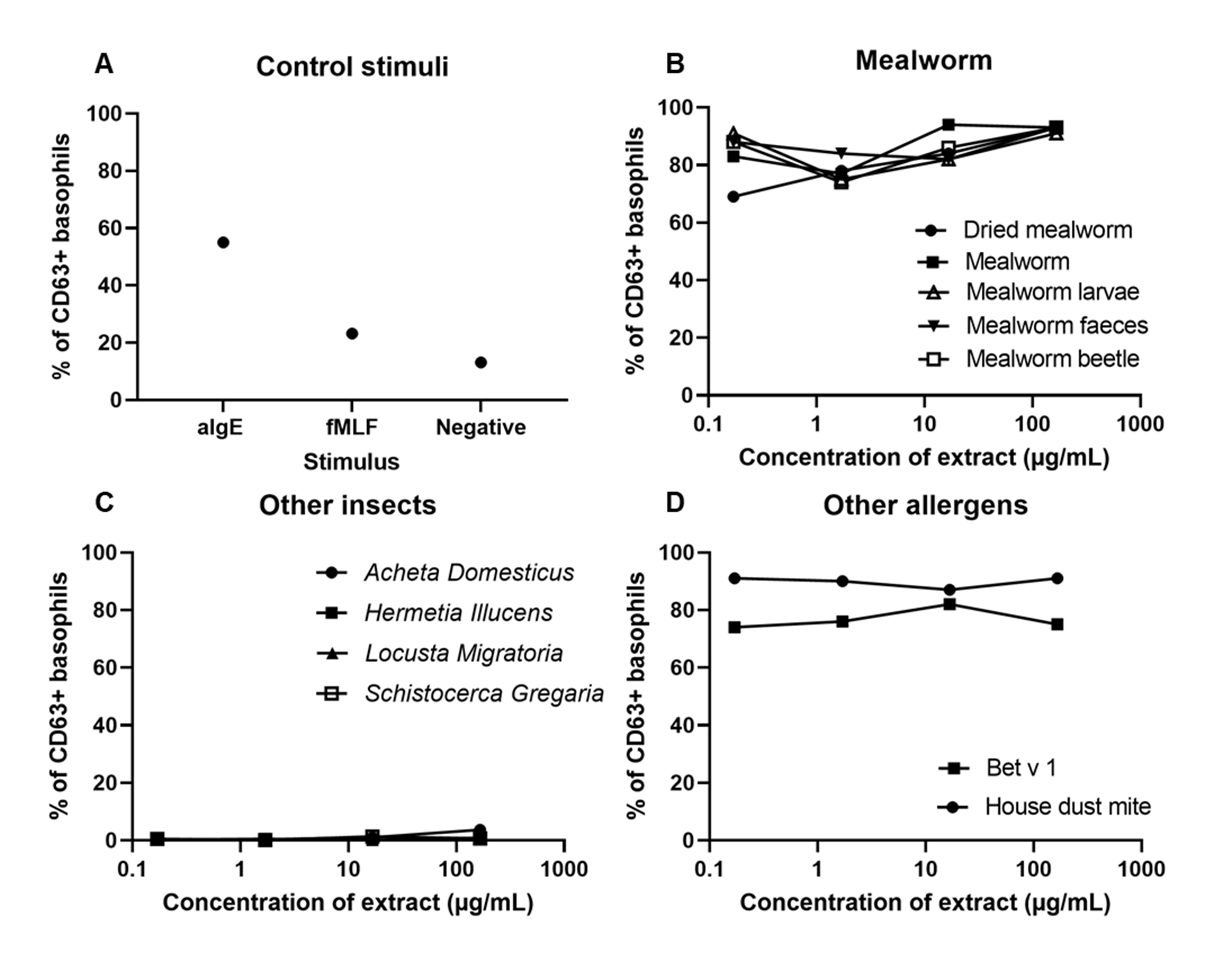

Supplement: Supplementary file 2 [file Figure_1_v1.tif]

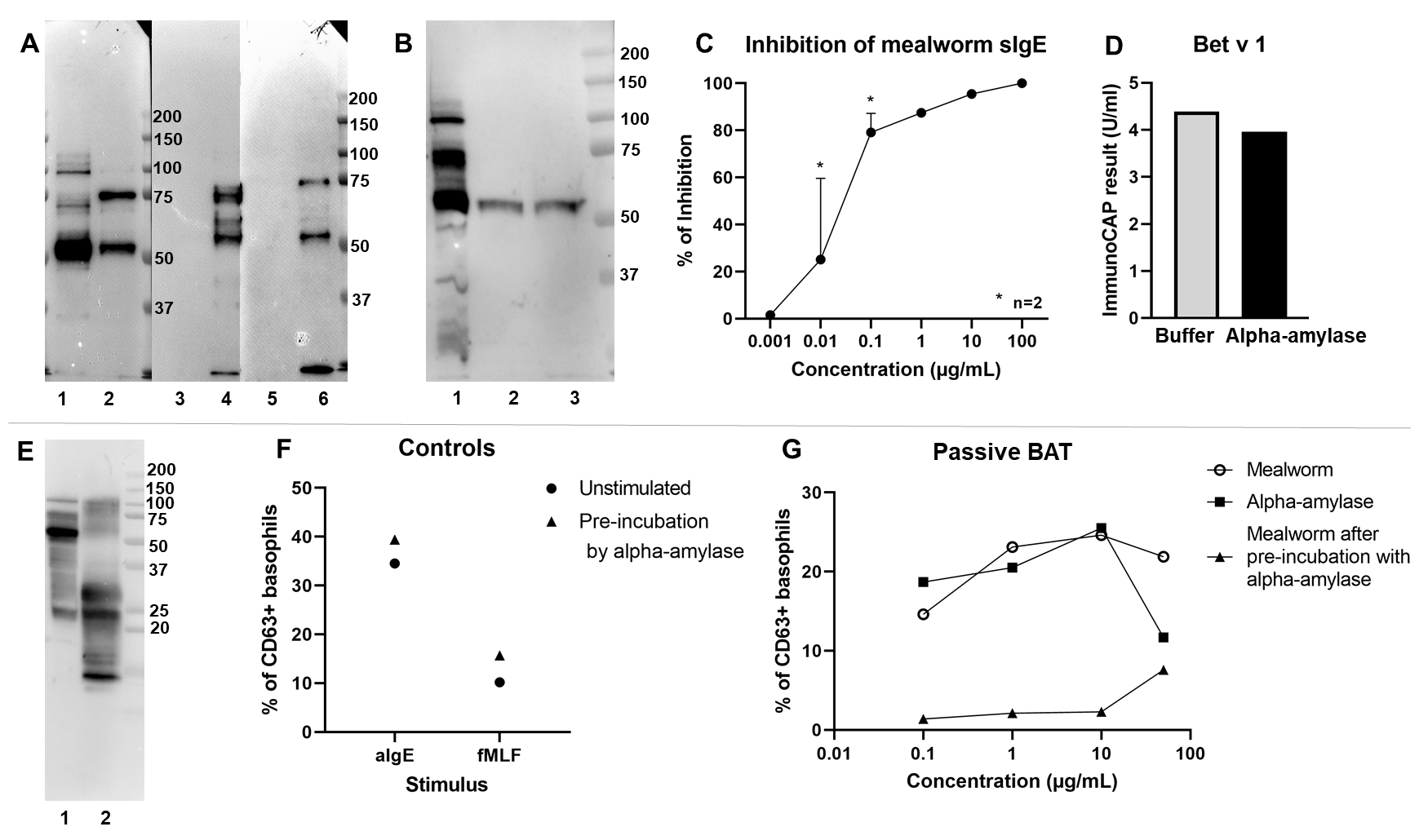

Supplement: Supplementary file 3 [file Figure_2_v1.tif]
